# Cardiovascular case fatality in rheumatoid arthritis is decreasing; first prospective analysis of a current low disease activity rheumatoid arthritis cohort and review of the literature

**DOI:** 10.1186/1471-2474-15-142

**Published:** 2014-04-29

**Authors:** Inger L Meek, Harald E Vonkeman, Mart AFJ van de Laar

**Affiliations:** 1 Arthritis Center Twente, University Twente and Medisch Spectrum Twente, 7500KA Enschede, Netherlands; 2 Department of Rheumatic diseases, UMC St Radboud, Huispost 470, postbus 9101, 6500 HB Nijmegen, The Netherlands

**Keywords:** Cardiovascular risk, Mortality, Rheumatoid arthritis

## Abstract

**Background:**

Previous studies found increased case fatality after myocardial infarction and more frequent sudden death in RA patients compared to non-RA subjects. The RA associated CV risk might be explained by the combined effects of chronic systemic inflammation and increased lifestyle associated cardiovascular risk factors, and modified by the use of medication such as non steroidal anti-inflammatory drugs, corticosteroids and disease modifying anti-rheumatic drugs. Trends in case fatality rate in RA after the introduction of potent anti-inflammatory biologic therapies and treat-to-target treatment strategies aiming at remission are not known. This study was performed to examine the cardiovascular fatality rate in current low disease activity RA, and to evaluate trends in RA associated CV case fatality over time.

**Methods:**

Prospective study to determine the incidence of fatal and nonfatal CV events in 480 RA patients included in the ACT-CVD cohort between February 2009 and December 2011. Patients with prior CV disease were excluded. Cox regression analysis was performed to determine CV event risk and contributing risk factors over time. The results of the cohort analysis were put into the context of a review of the literature to evaluate trends in RA associated CV fatality rate over time.

**Results:**

The study included 480 RA patients, 72.3% female with median disease duration of 4.2 years, 72.1% being in clinical remission (Disease Activity Score in 28 joints). During a mean follow up of 2.9 years 29 patients (6%) experienced a first CV event, 2 fatal and 27 non-fatal, corresponding to a 6.9% case fatality rate. Comparison with previous studies in cohorts with successive enrolment periods shows a trend towards a decrease in CV case fatality in RA from 52.9% in 1998 to 6.9% in our study.

**Conclusion:**

CV case fatality in current low disease activity RA is importantly lower than in previous studies, and a trend towards decreasing CV fatality in RA is suggested.

## Background

Rheumatoid arthritis (RA) is a chronic inflammatory disease involving multiple organ systems. RA is associated with a decreased life expectancy, and premature death is mainly due to accelerated atherosclerotic cardiovascular (CV) disease [1-4]. Recent meta-analyses confirmed that compared to the general population the overall increase of both CV disease and death in RA is approximately 50% [5-7]. CV disease in RA is more severe and associated with a worse prognosis, which was shown by studies in RA cohorts with enrollment in the 1990s that found significantly increased 30-day mortality after myocardial infarction and more frequent sudden death compared to non-RA patients [8,9].

In the general population of high income countries the patients’ prognosis after a CV event improved significantly during the second half of the 20th century. Thirty to fifty percent of the fall in general population CV mortality in this period can be attributed to improved survival after a CV event [10]. Analyses of time trends in RA associated CV events show conflicting results and also recent studies show increased CV events in RA patients compared to the general population [6,11,12].

It is generally thought that RA associated CV risk is the consequence of the combined effects of chronic systemic inflammation and increased traditional CV risk factors, and modified by the use non steroidal anti-inflammatory drugs (NSAIDs), corticosteroids and disease modifying anti-rheumatic drugs (DMARDs) [9,13-18]. After the introduction of potent anti-inflammatory biologic therapies and tight control treatment strategies into daily clinical care the degree of systemic inflammation and severity of physical disability have importantly improved. Therefore we performed this study to examine if the high risk of death in RA associated CV disease persists in tight controlled RA. We evaluated the incidence of fatal and non-fatal CV events in a cohort of established, currently low disease activity RA patients between February 2009 and December 2012 and calculated CV case fatality rates. Subsequently we put these results into the perspective of previous reports on mortality in RA associated CV disease to explore trends in RA associated CV case fatality over time.

## Methods

### Patients

The Arthritis Center Twente Cardiovascular Disease (ACT-CVD) project was established in 2009. The method of patient inclusion and baseline data collection in this cohort has been described previously [19]. Briefly, in 2009 the Arthritis Center Twente (ACT) in Enschede, the Netherlands, introduced a CV screening protocol as part of routine daily clinical practice. The ACT-CVD database contains the anonimised baseline demographics, CV risk factors and rheumatic disease characteristics of all, both prevalent and incident, participating patients. Individuals are classified according to their clinical diagnosis as registered by experienced attending rheumatologists. Disease duration was calculated as time from RA diagnosis until the CV screening visit. At the ACT RA disease activity is systematically measured by Disease Activity Score in 28 joints (DAS-28), RA remission being defined as a DAS-28 ≤ 2.6 [20]. After inclusion in the database, patients are followed up to a first CV event, death or censoring. Follow up data on incident CV events and causes of death are extracted from the hospital electronic registration system and are validated by medical chart review. For out of hospital events and death, attending general practitioners receive periodic questionnaires and data is extracted from the Dutch national registry of death certificates. Events are considered to be of CV origin when the diagnosis was confirmed by a cardiologist. For this study the data of all RA patients without prior CV disease in the ACT-CVD database that completed the CV screening protocol before December 2011 were used (all RA 508; included 480).

The protocol for data collection and storage in the ACT-CVD project was approved by the Arhtritis Center Twente Institutional Review Board. Because the study contains data from daily clinical practice the ethics committees determined, in accordance to Dutch law, that no approval was requiered. Nontheless, patients were fully informed and only the data of patients that gave informed consent were entered into the ACT-CVD database.

### Follow up and definition of CV events

All participants were followed up for the occurrence of fatal and non-fatal CV events. The definition ‘CV event’ included (1) myocardial infarction; (2) coronary intervention, i.e. percutaneous transluminal coronary angioplasty (PTCA) or coronary artery bypass graft (CABG); (3) angina pectoris; (4) acute heart failure; (5) cerebral vascular accident (CVA); (6) death due to cardiac causes; (7) sudden death. Sudden death was considered a CV event because it is generally from CV origin [21]. Duration of follow up was calculated as the interval between inclusion and first CV event or death, or censored at December 1st 2012, whichever came first.

### Literature review

The Medline database was searched from its inception to April 2013 for research articles and reviews published in English studying CV disease in RA. The search terms RA, cardiovascular, CVD, CVA, risk, co morbidity, mortality, and death were used alone or in combination. Reference lists of key publications were hand searched for additional references. We selected peer reviewed articles (cohort studies and meta-analyses) that met the following criteria: (1) predefined RA criteria; (2) inclusion of both male and female gender; (3) pre-specified CV disease criteria; (4) information on prior CV disease; (5) information on RA disease duration before inclusion; (6) information on events per patient year follow up. If data from a single study were reported in more than one article only the results from the most relevant publication were included in the literature review. Because a recent meta-analysis showed that inception cohorts were the only studies that did not find an increase of incident CV disease in RA, we considered inception and non-inception cohorts seperately [6].

### Statistical analysis

Baseline characteristics of the RA cohort and incidence of CV events were presented by appropriate descriptive statistics. COX regression analysis was performed to determine CV event risk and contributing risk factors over time. Statistical analyses were performed using IBM-SPSS statistics software version 20.0.

## Results

The ACT-CVD cohort included 1668 subjects, 508 with a diagnosis of RA. From these, 28 were excluded because of prior documented CV disease.

### Baseline characteristics of RA patients

The present study included 480 RA patients. The patients’ mean age was 59.0 years and 72.3% were women. Median RA disease duration was 4 years, 63% was IgM rheumatoid factor and/or anti-CCP positive and 42% had erosive joint disease. At inclusion in the ACT-CVD cohort, 390 (81%) patients were using anti-inflammatory immunosuppressive therapy; synthetic disease modifying anti-rheumatic drugs (DMARDs, 72%), biologicals (in majority tumour necrosis factor α inhibitor; any biological 23%, tumour necrosis factor α inhibitor 22%) or corticosteroids (14%), either alone or in combination. Mean disease activity was low, DAS-28 2.5, 72% of patients being in clinical remission (Table 1).

**Table 1:** Distributions of potential risk factors for occurrence of cardiovascular events in RA patients at baseline

	**N = 480**
***Demographics***	
Sex (n,% female)	347 (72.3)
Age (mean, SD)	59.0 (13.0)
***Traditional CV risk factors***	
Smoking, current (n,%)	114 (23.8)
Systolic blood pressure (mmHg, mean, SD)	144.0 (22.9)
Total cholesterol (mmol/L, SD)	5.3 (0.99)
LDL cholesterol (mmol/L, SD)	3.1 (0.83)
Triglycerides (mmol/L, SD)	1.3 (0.65)
Atherogenic index (mean, SD)	3.7 (1.1)
GlyHb (%, mean, SD)	5.8 (0.67)
SCORE 10-year estimated CV risk (%, SD))	5.7 (4.9)
***Inflammatory markers***	
ESR (mm/hr, mean, SD)	16.5 (14.9)
Hs CRP (mg/L, mean, SD)	7.0 (10.0)
***RA disease characteristics***	
RA disease duration (years; median, 25^th^-75^th^ percentile)	4.2 (1.5-11.3)
Seropositive (anti-CCP and/or IgMRF; n,%)	286 (63.3)
Erosions (n,%)	198 (42.2)
DAS 28 (mean, SD)	2.5 (1.2)
Remission (n,%)	223 (72.1)
***Medication***	
DMARD (n,%)	350 (72.9)
MTX (n,%)	291 (60.6)
TNFα inhibitor (n,%)	105 (21.9)
NSAID (n,%)	177 (36.9)
Corticosteroids (n,%)	68 (14.2)

(*RA*: rheumatoid arthritis; *CV*: cardiovascular; *SD*: standard deviation; *LDL*: low density lipoprotein; GlyHb: glycated hemoglobin; *ESR*: erythrocyte sedimentation rate; *Hs CRP*: high sensitivity C-reactive protein; anti CCP: anti cyclic citrullinated protein; *IgM RF*: *IgM* rheumatoid factor; DAS28: disease activity score in 28 joints; *DMARD*: disease modifying antirheumatic drug; *MTX*: methotrexate; *TNF* inhibitor: tumour necrosis factor α inhibitor; *NSAID*: non steroidal anti inflammatory drug).

### Incident CV events

During the follow up period, 29 patients (6%) experienced a first CV event. The mean follow up period was 2.9 years (SD 0.65) and total follow up of 1380 patient-years, resulting in a CV event rate of 21/1000 patient-years (95% CI 14.3-29.8). The different CV diagnoses are listed in Table 2. There were 2 fatal CV events and 27 non-fatal. From the ten cases first presenting with cardiac chest pain, three underwent a coronary intervention procedure and three experienced a second CV event during the total follow up period. None of the patients that experienced a non-fatal CV event died within the following 30 days, resulting in a 6.9% CV case fatality rate (Table 3 and Additional file 1: Table S1). COX regression analysis evaluating the relation between traditional CV risk factors, inflammatory parameters, RA disease duration, presence of IgM rheumatoid factor and/or anti-CCP antibodies and use of anti-inflammatory immunosuppressive therapy (i.e. methotrexate, non-methotrexate DMARDs, tumour necrosis factor α inhibitors or corticosteroids, which were considered seperately) and the occurrence of CV events showed only statistically significant independent risks of increasing systolic blood pressure (HR 1.016, 95% CI 1.002-1.030) and use of antihypertensive medications (HR 2.829, 95% CI 1.358-5.891) (Additional file 2: Table S2). The use of methotrexate was protective against incident first CV events (HR 3.436, 95% CI 1.553-7.576).

**Table 2:** Distributions of cardiovascular events in RA patients according to disease duration (<6 months: incident RA, ≥6 months: prevalent RA and IgM rheumatoid factor and/or anti-CCP positivity

**Event type (n,%)**	**RA (n = 480)**	**Incident RA (n = 60)**	**Prevalent RA (n = 240)**	**Seronegative RA (n = 166)**	**Seropositive RA (n = 286)**
Myocardial infarction	2 (6.9)	1 (33.3)	1 (3.8)	1 (10.0)	1 (5.9)
Acute coronary syndrome	10 (34.5)	2 (66.6)	9 (34.6)	4 (40.0)	7 (41.2)
Acute heart failure	4 (13.8)	0 (0.0)	4 (15.4)	1 (10.0)	2 (11.8)
Coronary intervention	5 (17.2)	0 (0.0)	5 (17.2)	2 (20.0)	2 (11.8)
Cerebrovascular accident	5 (17.2)	0 (0.0)	5 (17.2)	1 (10.0)	4 (23.5)
Cardiac death	2 (6.9)	0 (0.0)	2 (7.7)	1 (10.0)	1 (5.9)
All	29 (100)	3 (100)	26 (100)	10 (100)	17 (100)
All, events/1000 py	21	18	21	20	21

(*RA*: rheumatoid arthritis; py: patient years).

**Table 3:** Characteristics of the 6 studies included in the literature review

**Reference**	**Country**	**Enrolment period**	**Mean follow up (years)**	**Sample type**	**Inception cohort**	**RA definition**	**% female**	**Mean age at entry**	**Previous CVD excluded**	**CVD included**	**Outcome ascertainment**	**N**	**Person-years at risk**	**CV events (n)**	**CV events/ 1000 person-years**	**Fatal CV events (%)**
Del Rincon, 2001 [13]	USA	1996	0.9	Clinic based	No	ACR 1987	62.3	56*	Yes	MI, CVA, CV death	Medical record	236	204	7	34.3	28.6
Assous, 2007 [23]	France	1998-1999	5.4	Clinic based	No	ACR 1987	83.8	55	Yes	MI, CVA, CV death	Medical record	239	NAV	17	13	52.9
Solomon, 2006 [23]	USA	1999-2003	2.8	Population based	No	ICD code	71.1	NAV	No	MI, CVA, CV death	ICD code	25,385	70,612	1,042	14.8	41.2
Peters, 2009 [24]	Netherlands	2001-2002	2.7	Clinic based	No	ACR 1987	65	63	Yes	Coronary disease, CVA, CV and sudden death	Medical record	272	729	19	26.1	12.9**
Meek, 2013*** [19]	Netherlands	2009-2011	2.9	Clinic based	No	Clinical diagnosis	72.3	59	Yes	Coronary disease, CVA, CV and sudden death	Medical record	480	1380	29	21	6.9
Maradit-Kremers, 2005 [9]	USA	1955-1995	14.7	Population based	Yes	ACR 1987	73	58	Yes	MI, CABG, PTCA, AP, CV and sudden death	Medical record	603	8,672	109	13.0	23.8
Holmqvist, 2010 [25]	Sweden	1995-2006	4.1	Population based	Yes	ACR 1987	71.0	56.9	Yes	MI, AP, CABG, PTCA, CV death	Hospital discharge register	7,469	33,436	341	10.2	11.1

Studies are placed in order of cohort type (non-inception vs inception) and enrolment period. *RA*: rheumatoid arthritis; *CVD*: cardiovascular disease; *MI*: myocardial infarction; *CVA*: cerebrovascular incident; *CV*: cardiovascular; *CABG*: coronary artery bypass graft; *PTCA*: percutaneous transluminal coronary angioplasty; *AP*: angina pectoris; *NAV*: not available. * Median. ** non-published data received from the authors. ***data from the ACT-CVD cohort presented in htis article.

### Literature review

We identified 24 studies and 2 meta-analyses evaluating CV disease in RA, of which 15 studies reported patient-years of follow up. Only nine studies evaluated composite endpoints for CV disease and thus facilitated a comparison with our own data. From these nine studies, four reported on data from the same cohort. Only the article providing data most relevant to our research was selected. One study reported CV outcome after only one year of follow up, which we considered to be short to evaluate CV mortality, but was included because of it’s high methodological quality. Table 3 provides a summary of the characteristics and results of the six selected studies, as well as our own data [9,13,22-25]. There was considerable heterogeneity in study characteristics such as cohort type, sample size, RA disease duration and length of follow up. We distinguished two inception and five non-inception cohorts. The studies showed no trend in CV event rate over time. However, there was a trend towards decline in percentage fatal CV events within the composite CV disease outcome in both inception and non-inception cohorts with successive periods of enrolment.

## Discussion

### Summary of findings

This is the first study to search for trends in case fatality in RA. The prospective analysis of the ACT-CVD cohort shows that first CV events were common, even though arthritis was tight controlled and overt inflammatory activity was low. However, the observed percentage CV deaths within the composite of CV events was importantly lower than observed in studies performed in older cohorts with successive periods of enrolment, suggesting a trend towards decreased case fatality.

### Comparison with previous studies

Our finding of a composite CV event rate of 21/1000 patient-years does not differ importantly from earlier clinic based non-inception cohorts evaluating CV disease incidence in RA. From the 1970s onwards many studies have found increased CV disease in RA, which showed some variation in their exact estimates for incident RA associated CV morbidity and mortality depending on factors such as sample size, cohort type, disease duration and length of follow up [5,26,27]. In general, large community based and inception cohort studies found lower CV event risks than smaller clinic based studies or studies including patients with established RA. Two recent meta-analyses confirmed an approximately 50% overall increase of both CV disease and death in RA compared to the general population. RA associated CV disease incidence was stable in the observation periods of the included studies which ranged from 1955 to 2006 [5-7]. Increased CV disease in RA is thought to be caused by a combination of increased traditional CV risk factors and disease specific risks, probably most importantly chronic systemic inflammation [28,29]. Different large cohort studies in the late 20^th^ and early 21^st^ century showed that inflammatory disease activity and disability in RA could be importantly improved by tight control treatment strategies using traditional synthetic and/or novel biologic DMARDs [30-32]. These treatment strategies are currently implemented into daily clinical care. However, an important decline in RA associated CV disease has not yet been obeserved [6,11,12].

In the general population CV disease is declining, largely due to improved prevention by general health and life style interventions. General population CV mortality is declining even faster because of better treatment of atherosclerotic vascular disease and improved event survival [10,33]. As mentioned previously, studies on CV disease in RA patients found increased CV event incidence, but some studies also observed that RA patients had a worse prognosis with more frequent fatality of CV events. In our cohort the proportion fatal among the composite of CV events was low. Comparison of our results with previous studies in non-inception cohorts with successive periods of enrolment suggests a trend of decreasing CV case fatality in RA from 1996 until now (28.6% vs. 6.9% fatal CV events, Table 3). This finding is supported by the results of two studies in inception cohorts, showing stable composite CV event rates in successive enrolment periods but a lower proportion fatal CV events between 1995–2006 compared to 1955–1995 (11.1% vs. 23.8% fatal CV events, Table 3).

A relative decrease in fatal CV events in RA over the last decades could be explained in different ways. On the one hand, reduction of the RA specific risk factors can result in a more benign course of RA associated CV disease and a CV event prognosis more similar to the general population. As mentioned previously, systemic inflammatory activity is thought to be the most important RA specific risk factor [28,29]. The majority of the patients in our cohort were treated following a tight control strategy targeted at remission induction that has been shown to establish stable low disease activity [34]. Also corticosteroid usage, associated with increased CV disease in RA, was very low in our cohort [15]. On the other hand, one might speculate that because of increased awareness, RA associated CV disease is now recognised at an earlier stage. In 2009 the EULAR published the first recommendations for CV risk management in RA. However, recent research has shown that implementation of these recommendations into clinical practice has not yet been widely established [35-37]. Our cohort was initiated before the publication of the EULAR recommendations, and in the ACT CV risk factor screening was not followed by a per protocol intensified CV risk management.

### Strengths and limitations

This study has several limitations. The relatively short duration of follow up of our cohort does not allow evaluation of trends in CV event rate over time within the same population, and it may also cause an underestimation of fatal CV events. In a non-inception cohort such as the ACT-CVD cohort this will probably be of lesser importance, as CV disease in RA is thought to develop and accumulate with longer disease duration. Also, the literature review included two other non-inception studies with similar follow up duration in different periods of enrolment, which allows comparison of results. As mentioned previously, studies on RA vary importantly in study population characteristics, and this was also true in our comparison to the literature. The incidence of CV disease may vary with ethnicity and social status, which variables were not registered in our database. The ACT is situated in a rural region of the Netherlands, where the vast majority of the population is of Caucasian origin, and access to healthcare is guaranteed by the national healthcare insurance system. Only one of the studies included in the literature review mentioned ethnicity of the study population, which was over 90% white race, and no study described social status characteristics. The proportion seropositive RA, a possible indicator of more severe phenotype, varied between studies and was lowest in our cohort. This limitation may account in part for our findings, as our patients may in fact have had milder disease and thus a lower overall inflammatory burden; however, our cohort does not differ markedly from others recently reported on tight control treatment in RA [30,32]. Because we identified only a limited number of studies that reported composite and fatal CV outcome parameters which allowed comparison with our own data, selection bias should be considered when interpreting the literature review, but we were able to include data from cohorts that contributed importantly to the present knowledge on RA associated CV disease. Finally, our study was explorative, and cannot provide definite conlusions because we do not have control CV event data. The possible trend of decreasing CV case fatality in RA should be confirmed by further research comparing CV event and case fatality rates in RA patients to those in the general population in the same region and time period.

## Conclusion

In this study in a current cohort of low disease activity RA the observed percentage CV deaths within the composite of CV events was importantly lower than in previous studies with successive periods of enrolment. A trend towards decreasing CV fatality in RA is suggested and should be re-evaluated in cohort studies with control populations and longer follow up.

## Abbreviations

RA: Rheumatoid arthritis; CV: Cardiovascular; ACT-CVD: Arthritis Center Twente CardioVascular Disease cohort; NSAIDs: Non-steroidal anti-inflammatory drugs; DMARDs: Disease modifying anti-rheumatic drugs; ACT: Arthritis Center Twente; DAS-28: Disease activity score in 28 joints; PTCA: Percutaneous transluminal coronary angioplasty; CABG: Coronary artery bypass graft; CVA: Cerebrovascular accident; CVD: Cardiovascular disease; Anti-CCP: Anti cyclic citrullinated pepside antibody; SD: Standard deviation; LDL: Low density lipoprotein; GlyHb: Glycated hemoglobin; ESR: Erythrocyte sedimantation rate; Hs CRP: High sensitivity C-reactive protein; IgM RF: IgM rheumatoid factor; MTX: Methotrexate; TNF: Tumour necrosis factor; MI: Myocardial infarction; AP: Angina pectoris.

## Competing interests

All authors have completed the Unified Competing Interest form at http://www.icmje.org/coi_disclosure.pdf (available on request from the corresponding author) and declare: no support from any organisation for the submitted work; no financial relationships with any organisations that might have an interest in the submitted work in the previous three years, no other relationships or activities that could appear to have influenced the submitted work.

## Authors’ contributions

All authors have contributed significantly to this study. ILM and HEV were involved in all phases from the planning of the study to the writing of the manuscript. MAFJvdL contributed to the ACT-CVD cohort design and commented on the statistical analysis of the data, interpretation of the results, and preparation of the manuscript. All authors have given final approval of the version to be published.

## Pre-publication history

The pre-publication history for this paper can be accessed here:

http://www.biomedcentral.com/1471-2474/15/142/prepub

## Supplementary Material

Additional file 1: Table S1 Distributions of potential risk factors for occurrence of cardiovascular events in RA patients at baseline, according to RA duration (<6 months: incident RA; ≥6 months: prevalent RA). (RA: rheumatoid arthritis; CV: cardiovascular; SD: standard deviation; LDL: low density lipoprotein; GlyHb: glycated hemoglobin; ESR: erythrocyte sedimentation rate; Hs CRP: high sensitivity C-reactive protein; anti CCP: anti cyclic citrullinated protein; IgM RF: IgM rheumatoid factor; DAS28: disease activity score in 28 joints; DMARD: disease modifying antirheumatic drug; MTX: methotrexate; TNF inhibitor: tumour necrosis factor α inhibitor; NSAID: non steroidal anti inflammatory drug). *p<0.05 seronegative vs seropositive. Click here for file

Additional file 2: Table S2 Distributions of potential risk factors for occurrence of cardiovascular events in RA patients at baseline, according to presence of IgM and/or anti-CCP antibodies. (RA: rheumatoid arthritis; CV: cardiovascular; SD: standard deviation; LDL: low density lipoprotein; GlyHb: glycated hemoglobin; ESR: erythrocyte sedimentation rate; Hs CRP: high sensitivity C-reactive protein; anti CCP: anti cyclic citrullinated protein; IgM RF: IgM rheumatoid factor; DAS28: disease activity score in 28 joints; DMARD: disease modifying antirheumatic drug; MTX: methotrexate; TNF inhibitor: tumour necrosis factor α inhibitor; NSAID: non steroidal anti inflammatory drug). *p<0.05 seronegative vs seropositive. Click here for file

## References

[B1] Monson RR Hall AP Mortality among arthritics J Chronic Dis 1976 29 459 467 10.1016/0021-9681(76)90086-2 939801

[B2] Goodson NJ Wiles NJ Lunt M Barrett EM Silman AJ Symmons DP Mortality in early inflammatory polyarthritis: cardiovascular mortality is increased in seropositive patients Arthritis Rheum 2002 46 2010 2019 10.1002/art.10419 12209502

[B3] Goodson N Marks J Lunt M Symmons D Cardiovascular admissions and mortality in an inception cohort of patients with rheumatoid arthritis with onset in the 1980s and 1990s Ann Rheum Dis 2005 64 1595 1601 10.1136/ard.2004.034777 15843450 PMC1755282

[B4] Gonzalez A Maradit KH Crowson CS Nicola PJ Davis JM III Therneau TM Roger VL Gabriel SE The widening mortality gap between rheumatoid arthritis patients and the general population Arthritis Rheum 2007 56 3583 3587 10.1002/art.22979 17968923

[B5] Avina-Zubieta JA Choi HK Sadatsafavi M Etminan M Esdaile JM Lacaille D Risk of cardiovascular mortality in patients with rheumatoid arthritis: a meta-analysis of observational studies Arthritis Rheum 2008 59 1690 1697 10.1002/art.24092 19035419

[B6] Avina-Zubieta JA Thomas J Sadatsafavi M Lehman AJ Lacaille D Risk of incident cardiovascular events in patients with rheumatoid arthritis: a meta-analysis of observational studies Ann Rheum Dis 2012 71 1524 1529 10.1136/annrheumdis-2011-200726 22425941

[B7] Levy L Fautrel B Barnetche T Schaeverbeke T Incidence and risk of fatal myocardial infarction and stroke events in rheumatoid arthritis patients. A systematic review of the literature Clin Exp Rheumatol 2008 26 673 679 18799105

[B8] Van Doornum S Brand C King B Sundararajan V Increased case fatality rates following a first acute cardiovascular event in patients with rheumatoid arthritis Arthritis Rheum 2006 54 2061 2068 10.1002/art.21932 16802340

[B9] Maradit-Kremers H Crowson CS Nicola PJ Ballman KV Roger VL Jacobsen SJ Gabriel SE Increased unrecognized coronary heart disease and sudden deaths in rheumatoid arthritis: a population-based cohort study Arthritis Rheum 2005 52 402 411 10.1002/art.20853 15693010

[B10] Smolina K Wright FL Rayner M Goldacre MJ Determinants of the decline in mortality from acute myocardial infarction in England between 2002 and 2010: linked national database study BMJ 2012 344 d8059 10.1136/bmj.d8059 22279113 PMC3266430

[B11] Krishnan E Lingala VB Singh G Declines in mortality from acute myocardial infarction in successive incidence and birth cohorts of patients with rheumatoid arthritis Circulation 2004 110 1774 1779 10.1161/01.CIR.0000142864.83780.81 15381644

[B12] Bergstrom U Jacobsson LT Turesson C Cardiovascular morbidity and mortality remain similar in two cohorts of patients with long-standing rheumatoid arthritis seen in 1978 and 1995 in Malmo, Sweden Rheumatology (Oxford) 2009 48 1600 1605 10.1093/rheumatology/kep301 19858122

[B13] Del Rincon I Williams K Stern MP Freeman GL Escalante A High incidence of cardiovascular events in a rheumatoid arthritis cohort not explained by traditional cardiac risk factors Arthritis Rheum 2001 44 2737 2745 10.1002/1529-0131(200112)44:12<2737::AID-ART460>3.0.CO;2-# 11762933

[B14] Suissa S Bernatsky S Hudson M Antirheumatic drug use and the risk of acute myocardial infarction Arthritis Rheum 2006 55 531 536 10.1002/art.22094 16874796

[B15] Avina-Zubieta JA Abrahamowicz M De Vera MA Choi HK Sayre EC Rahman MM Sylvestre MP Wynant W Esdaile JM Lacaille D Immediate and past cumulative effects of oral glucocorticoids on the risk of acute myocardial infarction in rheumatoid arthritis: a population-based study Rheumatology (Oxford) 2013 52 68 75 10.1093/rheumatology/kes353 23192907

[B16] Solomon DH Kremer J Curtis JR Hochberg MC Reed G Tsao P Farkouh ME Setoguchi S Greenberg JD Explaining the cardiovascular risk associated with rheumatoid arthritis: traditional risk factors versus markers of rheumatoid arthritis severity Ann Rheum Dis 2010 69 1920 1925 10.1136/ard.2009.122226 20444756 PMC2963658

[B17] Solomon DH Glynn RJ Rothman KJ Schneeweiss S Setoguchi S Mogun H Avorn J Stürmer T Subgroup analyses to determine cardiovascular risk associated with nonsteroidal antiinflammatory drugs and coxibs in specific patient groups Arthritis Rheum 2008 59 1097 1104 10.1002/art.23911 18668605 PMC2884183

[B18] Peters MJ Van Sijl AM Voskuyl AE Sattar N Smulders YM Nurmohamed MT The effects of tumor necrosis factor inhibitors on cardiovascular risk in rheumatoid arthritis Curr Pharm Des 2012 18 1502 1511 10.2174/138161212799504786 22364134

[B19] Meek IL Picavet HS Vonkeman HE Verschuren WM van de Laar MA Increased cardiovascular risk factors in different rheumatic diseases compared with the general population Rheumatology (Oxford) 2013 52 210 216 10.1093/rheumatology/kes194 22847678

[B20] van Gestel AM Haagsma CJ van Riel PL Validation of rheumatoid arthritis improvement criteria that include simplified joint counts Arthritis Rheum 1998 41 1845 1850 10.1002/1529-0131(199810)41:10<1845::AID-ART17>3.0.CO;2-K 9778226

[B21] Kannel WB Plehn JF Cupples LA Cardiac failure and sudden death in the Framingham Study Am Heart J 1988 115 869 875 10.1016/0002-8703(88)90891-5 3354416

[B22] Assous N Touze E Meune C Kahan A Allanore Y Cardiovascular disease in rheumatoid arthritis: single-center hospital-based cohort study in France Joint Bone Spine 2007 74 66 72 10.1016/j.jbspin.2006.10.001 17174586

[B23] Solomon DH Avorn J Katz JN Weinblatt ME Setoguchi S Levin R Schneeweiss S Immunosuppressive medications and hospitalization for cardiovascular events in patients with rheumatoid arthritis Arthritis Rheum 2006 54 3790 3798 10.1002/art.22255 17136752

[B24] Peters MJ Van Halm V Voskuyl AE Smulders YM Boers M Lems WF Visser M Stehouwer CD Dekker JM Nijpels G Heine R Dijkmans BA Nurmohamed MT Does rheumatoid arthritis equal diabetes mellitus as an independent risk factor for cardiovascular disease? A prospective study Arthritis Rheum 2009 61 1571 1579 10.1002/art.24836 19877093

[B25] Holmqvist ME Wedren S Jacobsson LT Klareskog L Nyberg F Rantapaa-Dahlqvist S Alfreddson L Askling J Rapid increase in myocardial infarction risk following diagnosis of rheumatoid arthritis amongst patients diagnosed between 1995 and 2006 J Intern Med 2010 268 578 585 10.1111/j.1365-2796.2010.02260.x 20698926

[B26] Ward MM Recent improvements in survival in patients with rheumatoid arthritis: better outcomes or different study designs? Arthritis Rheum 2001 44 1467 1469 10.1002/1529-0131(200106)44:6<1467::AID-ART243>3.0.CO;2-6 11407710

[B27] Ward MM Interpreting studies of cardiovascular mortality in rheumatoid arthritis: the importance of timing Arthritis Rheum 2008 59 1687 1689 10.1002/art.24170 19035434 PMC2766778

[B28] Libby P Role of inflammation in atherosclerosis associated with rheumatoid arthritis Am J Med 2008 121 S21 S31 10.1016/j.amjmed.2008.06.014 18926166

[B29] Sattar N McCarey DW Capell H McInnes IB Explaining how “high-grade” systemic inflammation accelerates vascular risk in rheumatoid arthritis Circulation 2003 108 2957 2963 10.1161/01.CIR.0000099844.31524.05 14676136

[B30] Klarenbeek NB Guler-Yuksel M van der Kooij SM Han KH Ronday HK Kerstens PJ Seys PE Huizinga TW Dijkmans BA Allaart CF The impact of four dynamic, goal-steered treatment strategies on the 5-year outcomes of rheumatoid arthritis patients in the BeSt study Ann Rheum Dis 2011 70 1039 1046 10.1136/ard.2010.141234 21415052

[B31] Grigor C Capell H Stirling A McMahon AD Lock P Vallance R Kincaid W Porter D Effect of a treatment strategy of tight control for rheumatoid arthritis (the TICORA study): a single-blind randomised controlled trial Lancet 2004 364 263 269 10.1016/S0140-6736(04)16676-2 15262104

[B32] Schipper LG Vermeer M Kuper HH Hoekstra MO Haagsma CJ Den Broeder AA van Riel P Fransen J van de Laar MA A tight control treatment strategy aiming for remission in early rheumatoid arthritis is more effective than usual care treatment in daily clinical practice: a study of two cohorts in the Dutch Rheumatoid Arthritis Monitoring registry Ann Rheum Dis 2012 71 845 850 10.1136/annrheumdis-2011-200274 22210852

[B33] O’Flaherty M Buchan I Capewell S Contributions of treatment and lifestyle to declining CVD mortality: why have CVD mortality rates declined so much since the 1960s? Heart 2013 99 159 162 10.1136/heartjnl-2012-302300 22962283

[B34] Vermeer M Kuper HH Moens HJ Drossaers-Bakker KW Van Der Bijl AE Van Riel PL van de Laar MA Sustained beneficial effects of a protocolized treat-to-target strategy in very early rheumatoid arthritis: Three year results of the DREAM remission induction cohort Arthritis Care Res (Hoboken ) 2013 65 8 1219 1226 10.1002/acr.21984 23436821

[B35] Peters MJ Symmons DP McCarey D Dijkmans BA Nicola P Kvien TK McInnes IB Haentzschel H Gonzalez-Gay MA Provan S Semb A Sidiropoulos P Kitas G Smulders YM Soubrier M Szekanecz Z Sattar N Nurmohamed MT EULAR evidence-based recommendations for cardiovascular risk management in patients with rheumatoid arthritis and other forms of inflammatory arthritis Ann Rheum Dis 2010 69 325 331 10.1136/ard.2009.113696 19773290

[B36] Bartels CM Kind AJ Thorpe CT Everett CM Cook RJ McBride PE Smith MA Lipid testing in patients with rheumatoid arthritis and key cardiovascular-related comorbidities: a medicare analysis Semin Arthritis Rheum 2012 42 9 16 10.1016/j.semarthrit.2012.01.005 22424813 PMC3404199

[B37] Desai SS Myles JD Kaplan MJ Suboptimal cardiovascular risk factor identification and management in patients with rheumatoid arthritis: a cohort analysis Arthritis Res Ther 2012 14 R270 10.1186/ar4118 23237607 PMC3674627

