# Supplementary material for: Cardiovascular case fatality in rheumatoid arthritis is decreasing; first prospective analysis of a current low disease activity rheumatoid arthritis cohort and review of the literature
Source: BMC Musculoskelet Disord. 2014 Apr 29;15:142. doi: 10.1186/1471-2474-15-142 (PMC4046075; doi:10.1186/1471-2474-15-142)
Supplement: Additional file 1: Table S1 — Distributions of potential risk factors for occurrence of cardiovascular events in RA patients at baseline, according to RA duration (<6 months: incident RA; ≥6 months: prevalent RA). (RA: rheumatoid arthritis; CV: cardiovascular; SD: standard deviation; LDL: low density lipoprotein; GlyHb: glycated hemoglobin; ESR: erythrocyte sedimentation rate; Hs CRP: high sensitivity C-reactive protein; anti CCP: anti cyclic citrullinated protein; IgM RF: IgM rheumatoid factor; DAS28: disease activity score in 28 joints; DMARD: disease modifying antirheumatic drug; MTX: methotrexate; TNF inhibitor: tumour necrosis factor α inhibitor; NSAID: non steroidal anti inflammatory drug). *p<0.05 seronegative vs seropositive. [file 1471-2474-15-142-S1.doc]

|  | **All**  **(n=480)** | **Incident RA**  **(n=60)** | **Prevalent RA**  **(n=420)** |
| --- | --- | --- | --- |
| ***Demographics*** |  |  |  |
| Sex (n, % female) | 347 (72.3) | 40 (66.7) | 307 (73.1) |
| Age (mean, SD)* | 59.0 (13.0) | 55.7 (11.9) | 59.45 (13.1) |
| ***Traditional CV risk factors*** |  |  |  |
| Smoking , current (n, %)* | 114 (23.8) | 23 (38.3) | 91 (21.7) |
| Systolic blood pressure (mmHg, mean, SD) | 144.0 (22.9) | 146.7 (24.3) | 143.6 (22.7) |
| Total cholesterol (mmol/L, SD) | 5.3 (0.99) | 5.1 (1.12) | 5.3 (0.97) |
| LDL cholesterol (mmol/L, SD) | 3.1 (0.83) | 3.1 (0.92) | 3.1 (0.82) |
| Triglycerids (mmol/L, SD) | 1.3 (0.65) | 1.3 (0.67) | 1.3 (0.65) |
| Atherogenic index (mean, SD) | 3.7 (1.1) | 3.9 (1.33) | 3.6 (1.07) |
| GlyHb (%, mean, SD) | 5.8 (0.67) | 5.8 (0.56) | 5.8 (0.68) |
|  |  |  |  |
| SCORE 10-year estimated CV risk (%, SD)) | 5.7 (4.9) | 5.4(4.6) | 5.8 (5.0) |
|  |  |  |  |
| ***Inflammatory markers*** |  |  |  |
| ESR (mm/hr, mean, SD)* | 16.5 (14.9) | 23.9 (18.6) | 15.5 (14.0) |
| Hs CRP (mg/L, mean, SD)* | 7.0 (10.0) | 10.6 (10.8) | 6.5 (9.8) |
|  |  |  |  |
| ***RA disease characteristics*** |  |  |  |
| RA disease duration (years; median, 25^th^-75^th^ percentile)* | 4.2 (1.5-11.3) | 0.0 (0.0-0.2) | 5.3 (2.5-13.8) |
| Seropositive (anti-CCP and/or IgMRF; n, %) | 286 (63.3) | 39 (68.4) | 247 (62.5) |
| Erosions (n, %)* | 198 (42.2) | 13 (23.2) | 185 (44.8) |
| DAS 28 (mean, SD)* | 2.5 (1.2) | 3.7 (1.2) | 2.4 (1.2) |
| Remission (n, %)* | 223 (72.1) | 16 (28.6) | 322 (78.0) |
|  |  |  |  |
| ***Medication*** |  |  |  |
| DMARD (n, %)* | 350 (72.9) | 22 (36.7) | 328 (78.1) |
| MTX (n, %)* | 291 (60.6) | 22 (36.7) | 276 (65.7) |
| TNFα inhibitor (n, %)* | 105 (21.9) | 2 (3.3) | 103 (24.5) |
| NSAID (n, %) | 177 (36.9) | 20 (33.3) | 157 (37.4) |
| Coricosteroids (n, %) | 68 (14.2) | 6 (10.0) | 62 (14.8) |

**Table S1.**  **Distributions of potential risk factors for occurrence of cardiovascular events in RA patients at baseline, according to RA duration (<6 months: incident RA; ≥6 months: prevalent RA).** (RA: rheumatoid arthritis; CV: cardiovascular; SD: standard deviation; LDL: low density lipoprotein; GlyHb: glycated hemoglobin; ESR: erythrocyte sedimentation rate; Hs CRP: high sensitivity C-reactive protein; anti CCP: anti cyclic citrullinated protein; IgM RF: IgM rheumatoid factor; DAS28: disease activity score in 28 joints; DMARD: disease modifying antirheumatic drug; MTX: methotrexate; TNF inhibitor: tumour necrosis factor α inhibitor; NSAID: non steroidal anti inflammatory drug). *p<0.05 seronegative vs seropositive.
