# Supplementary material for: Cardiovascular case fatality in rheumatoid arthritis is decreasing; first prospective analysis of a current low disease activity rheumatoid arthritis cohort and review of the literature
Source: BMC Musculoskelet Disord. 2014 Apr 29;15:142. doi: 10.1186/1471-2474-15-142 (PMC4046075; doi:10.1186/1471-2474-15-142)
Supplement: Additional file 2: Table S2 — Distributions of potential risk factors for occurrence of cardiovascular events in RA patients at baseline, according to presence of IgM and/or anti-CCP antibodies. (RA: rheumatoid arthritis; CV: cardiovascular; SD: standard deviation; LDL: low density lipoprotein; GlyHb: glycated hemoglobin; ESR: erythrocyte sedimentation rate; Hs CRP: high sensitivity C-reactive protein; anti CCP: anti cyclic citrullinated protein; IgM RF: IgM rheumatoid factor; DAS28: disease activity score in 28 joints; DMARD: disease modifying antirheumatic drug; MTX: methotrexate; TNF inhibitor: tumour necrosis factor α inhibitor; NSAID: non steroidal anti inflammatory drug). *p<0.05 seronegative vs seropositive. [file 1471-2474-15-142-S2.doc]

|  | **All**  **(n=480)** | **Seronegative RA (n=166)** | **Seropositive RA (n=286)** |
| --- | --- | --- | --- |
| ***Demographics*** |  |  |  |
| Sex (n, % female) | 347 (72.3) | 119 (71.7) | 207 (72.4) |
| Age (mean, SD) | 59.0 (13.0) | 60.0 (13.5) | 58.6 (12.3) |
| ***Traditional CV risk factors*** |  |  |  |
| Smoking , current (n, %)* | 114 (23.8) | 25 (15.1) | 84 (29.4) |
| Systolic blood pressure (mmHg, mean, SD) | 144.0 (22.9) | 144.3 (24.2) | 143.5 (22.3) |
| Total cholesterol (mmol/L, SD) | 5.3 (0.99) | 5.3 (1.08) | 5.3 (0.93) |
| LDL cholesterol (mmol/L, SD) | 3.1 (0.83) | 3.1 (0.89) | 3.2 (0.80) |
| Triglycerids (mmol/L, SD) | 1.3 (0.65) | 1.4 (0.76) | 1.3 (0.58) |
| Atherogenic index (mean, SD) | 3.7 (1.1) | 3.7 (1.21) | 3.7 (1.07) |
| GlyHb (%, mean, SD) | 5.8 (0.67) | 5.9 (0.70) | 5.8 (0.65) |
|  |  |  |  |
| SCORE 10-year estimated CV risk (%, SD)) | 5.7 (4.9) | 5.7 (5.1) | 5.7 (4.8) |
|  |  |  |  |
| ***Inflammatory markers*** |  |  |  |
| ESR (mm/hr, mean, SD)* | 16.5 (14.9) | 13.3 (13.8) | 18.5 (15.5) |
| Hs CRP (mg/L, mean, SD) | 7.0 (10.0) | 6.7 (9.6) | 7.0 (10.1) |
|  |  |  |  |
| ***RA disease characteristics*** |  |  |  |
| RA disease duration (years; median, 25^th^-75^th^ percentile) | 4.2 (1.5-11.3) | 3.3 (1.2-7.3) | 4.8 (1.8-12.8) |
| Seropositive (anti-CCP and/or IgMRF; n, %) | 286 (63.3) | n.a. | n.a. |
| Erosions (n, %)* | 198 (42.2) | 36 (22.4) | 150 (53.2) |
| DAS 28 (mean, SD) | 2.5 (1.2) | 2.4 (1.2) | 2.6 (1.2) |
| Remission (n, %) | 223 (72.1) | 121 (75.2) | 198 (70.2) |
|  |  |  |  |
| ***Medication*** |  |  |  |
| DMARD (n, %) | 350 (72.9) | 123 (74.1) | 211 (73.8) |
| MTX (n, %)* | 291 (60.6) | 94 (56.6) | 190 (66.4) |
| TNFα inhibitor (n, %) | 105 (21.9) | 32 (19.3) | 65 (22.7) |
| NSAID (n, %) | 177 (36.9) | 62 (37.3) | 108 (37.8) |
| Coricosteroids (n, %) | 68 (14.2) | 22 (13.3) | 40 (14.0) |

**Table S2.** **Distributions of potential risk factors for occurrence of cardiovascular events in RA patients at baseline, according to presence of IgM and/or anti-CCP antibodies.** (RA: rheumatoid arthritis; CV: cardiovascular; SD: standard deviation; LDL: low density lipoprotein; GlyHb: glycated hemoglobin; ESR: erythrocyte sedimentation rate; Hs CRP: high sensitivity C-reactive protein; anti CCP: anti cyclic citrullinated protein; IgM RF: IgM rheumatoid factor; DAS28: disease activity score in 28 joints; DMARD: disease modifying antirheumatic drug; MTX: methotrexate; TNF inhibitor: tumour necrosis factor α inhibitor; NSAID: non steroidal anti inflammatory drug). *p<0.05 seronegative vs seropositive.
